# Dysregulated immune system networks in war veterans with PTSD is an outcome of altered miRNA expression and DNA methylation

**DOI:** 10.1038/srep31209

**Published:** 2016-08-11

**Authors:** Marpe Bam, Xiaoming Yang, Elizabeth E. Zumbrun, Yin Zhong, Juhua Zhou, Jay P. Ginsberg, Quinne Leyden, Jiajia Zhang, Prakash S. Nagarkatti, Mitzi Nagarkatti

**Affiliations:** 1 Department of Pathology, Microbiology and Immunology, University of South Carolina School of Medicine, Columbia, SC 29209, USA; 2 William Jennings Bryan Dorn Veterans Medical Center, 6439 Garners Ferry Road, Columbia, 29209-1639, South Carolina, USA; 3 Department of Epidemiology and Biostatistics, Arnold School of Public Health, University of South Carolina, Columbia, SC 29206, USA

## Abstract

Post-traumatic stress disorder patients experience chronic systemic inflammation. However, the molecular pathways involved and mechanisms regulating the expression of genes involved in inflammatory pathways in PTSD are reported inadequately. Through RNA sequencing and miRNA microarray, we identified 326 genes and 190 miRNAs that were significantly different in their expression levels in the PBMCs of PTSD patients. Expression pairing of the differentially expressed genes and miRNAs indicated an inverse relationship in their expression. Functional analysis of the differentially expressed genes indicated their involvement in the canonical pathways specific to immune system biology. DNA methylation analysis of differentially expressed genes also showed a gradual trend towards differences between control and PTSD patients, again indicating a possible role of this epigenetic mechanism in PTSD inflammation. Overall, combining data from the three techniques provided a holistic view of several pathways in which the differentially expressed genes were impacted through epigenetic mechanisms, in PTSD. Thus, analysis combining data from RNA-Seq, miRNA array and DNA methylation, can provide key evidence about dysregulated pathways and the controlling mechanism in PTSD. Most importantly, the present study provides further evidence that inflammation in PTSD could be epigenetically regulated.

PTSD develops after exposure to a traumatic event, such as military combat, violence, natural disaster, and the like. While in the US general population alone, the prevalence rate of PTSD is estimated to be ~3.5%, the rate increases significantly to ~20% in U.S. military service personnel, following combat^1^,^2^ leading to an estimated annual healthcare cost of around 180 million US dollars^3^. Moreover, the symptoms may last for a very long time, which significantly affects the quality of life. PTSD is a serious psychiatric disorder with a poor understanding of its etiology, especially at the molecular level. Symptoms of PTSD include hyperarousal, intrusive thoughts, flashbacks, nightmares, numbing of feelings, insomnia, fear, avoidance of reminders, irritability, hypervigilance, heightened startle response and distress when exposed to reminders^4^. It is believed that numerous molecular factors determine the risk and subsequent development of PTSD^5^ and such studies are now at the forefront of molecular psychiatry research. Several genes has been reported to be differentially expressed in PTSD which led to the identification of dysregulated immune system causing inflammation in the patients^6^,^7^,^8^.

A recent review analyzed published literature on immune status and concluded that PTSD patients exhibit excessive inflammatory state^9^. Interestingly, PTSD has also been associated with other clinical disorders such as cardiovascular disease, diabetes, gastrointestinal disease, fibromyalgia, chronic fatigue syndrome, musculoskeletal disorders, and autoimmune diseases^10^,^11^, all of which have an inflammatory component. Thus, it is important to study the molecular changes occurring in the immune cells and the inflammation manifested in PTSD patients. Peripheral blood mononuclear cells (PBMCs), which include T cells, B cells and monocytes are major players in the peripheral immune system and constitute cells that are both pro- and anti-inflammatory in nature. Additionally, many of the cytokines and chemokines of the pro- and anti-inflammatory milieu are produced by the PBMCs and can give rise to profound changes in the immune response^12^,^13^,^14^. The consensus now is that, even though PTSD is a psychiatric disorder, PBMCs play a major role in exacerbating the symptoms^15^,^16^. However, the knowledge of initiation of inflammation and the canonical pathways dysregulated during PTSD in PBMCs are poorly understood.

Recent use of powerful techniques like Next Generation Sequencing (NGS) and microarrays has led to the identification of differentially expressed mRNAs and miRNAs at the global level. Moreover, combining data from these techniques with various bioinformatics tools for data analysis has made it possible to predict and discern biological pathways that are affected in a diseased state, thereby making it possible to overcome the limitations of single gene-based studies. Furthermore, data from miRNA expression arrays and DNA methylation studies can be used to predict and study the regulation of expression of differentially expressed genes at the epigenetic level. Epigenetic regulation of gene expression includes the influence from processes like DNA methylation, histone modifications and miRNA expression^17^,^18^,^19^. Micro-RNAs are small, ~22 nucleotide long, non-coding, regulatory RNAs and are one of the key epigenetic entities that regulate the expression of genes at the post-transcriptional level^20^. The mechanism of gene regulation by miRNAs involve physical interactions with complimentary sequences, typically at the 3′ untranslated regions (UTR) of an mRNA leading to the degradation of the mRNA or inhibition of translation^21^,^22^. Recently, in mammals, it was reported that most (66%–>90%) of the mRNA-miRNA interaction leads to destabilization of the mRNA^23^, clearly implying that differential expression of miRNAs can lead to change in the level of transcripts of gene(s). Regulation of numerous immune system genes by miRNAs has already been reported demonstrating that the immune system is tightly regulated by the miRNAs^6^,^7^,^24^,^25^,^26^. For example, our lab has shown that elevated expression of interferon gamma (IFNG) in PTSD is regulated by hsa-miR-125a, which is found to be downregulated in PTSD patients^6^. In a related study, we have shown that expression of another pro-inflammatory cytokine, interleukin (IL)-12, is elevated in PTSD and its expression is correlated with downregulation of hsa-miR-193a-5p^7^ and many other miRNAs (data not shown). On the other hand, unlike miRNAs, DNA methylation at CpG islands present near the transcription start sites (TSS) can regulate the expression of a gene at the transcriptional level^27^,^28^,^29^. Effects brought about by DNA methylation is also reported to influence the expression of several genes including immune system related genes^30^,^31^. Until now, the majority of the studies intended to identify gene regulators have focused on a single or few genes/miRNAs at a time. Moreover, to our knowledge there is no report on expression paired analysis using RNA-Seq and miRNA array data to correlate differentially expressed genes in PTSD. Therefore, combining RNA-Seq and miRNA array data is a novel approach to simultaneously identify several genes and their biological pathways that are dysregulated and their regulators (miRNAs, in this case) that are possibly causing the differential expression of the genes.

In the present study, we could identify and predict many differentially expressed genes involved in canonical pathways related to the immune system biology, in the PBMCs of PTSD patients. We further provide preliminary evidence at the global level that the differential expression of the genes is possibly an outcome of differential expression of miRNAs and change in DNA methylation level.

## Results

### Gene expression analysis in PTSD reveals differentially expressed genes

**Figure 1:**
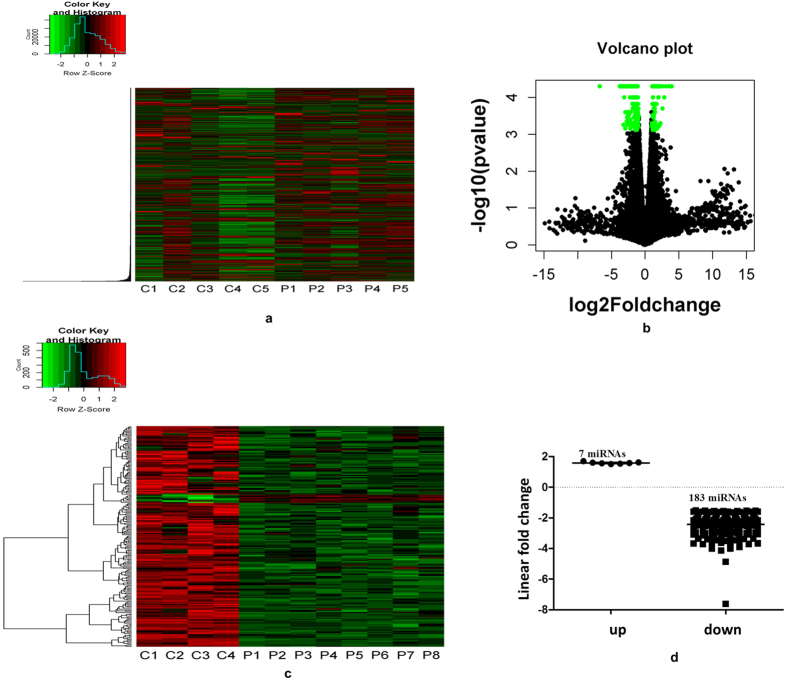
RNA-seq and miRNA microarray reveal differentially expressed genes and miRNAs in PTSD. **(a)** Heat map showing the expression level of genes after RNA-Seq analysis (C: Control and P: PTSD patient). Five individuals each in control and PTSD groups were included for RNA-Seq analysis. **(b)** Volcano plot showing genes with log2 fold change of at least 1 and *p* value of at least 0.005. We obtained 326 protein coding Ids and 40 non-coding RNA Ids with this criteria. **(c)** Heat map showing miRNA expression levels after microarray performed with total RNA from 4 controls and 8 PTSD patients. (**d)** The graph shows 190 miRNAs that were differentially expressed (*p *≤ 0.05 and at least 1.5 linear fold change, 7 up- and 183 down-regulated). The positioning of the miRNAs on the graph is on the basis of their linear fold changes of the expression values.

We performed RNA-Seq on RNA samples from PBMCs of PTSD patients (Table 1 provides the demographics of the controls and the patients included for the RNA-Seq analysis) and, on average, obtained ~60 million reads per sample which resulted in a good sequencing depth upon considering the size of human genome. A total of 40420 mRNA and 11218 non-coding RNA Ids were present in the list after obtaining the RNA-Seq data (Fig. 1a) and 48518 Ids were obtained after initial quality control. The list after quality control included both coding and non-coding RNAs as well as their transcript/splice variants, due to which the number was possibly very high for PBMCs. We selected genes keeping a cutoff value of 2 or more fold change plus a p value < 0.005 and obtained a total of 326 mRNAs (Fig. 1b) and 40 non-coding RNAs which were significantly changed in their expression levels based on RNA-Seq analysis (Supplementary Table S3 provides the list of the differentially expressed Ids). Of the 326 mRNAs, 64 (19.63%) were up-regulated and 262 (80.36%) downregulated (here, the differentially expressed genes included only the protein coding genes and not the non-coding Ids which made a significant proportion of the total Ids obtained after RNA-Seq data analysis). Since miRNAs are the main entities in this study, we looked for possible alterations in the transcript level of primary and pre-miRNAs. None of the significantly differentially expressed non-coding Ids included primary or pre-miRNAs. With regards to mature miRNAs, it is understood that due to their small size (~22 nucleotides), they will not be included during size selection in the RNA-Seq library preparation stage stage.

### Micro-RNA expression analysis indicates a global downregulation in PTSD

Table 1 provides the demographics of the controls and the patients included for the miRNA microarray analysis. At the time of performing the miRNA array, 847 probes for miRNAs were used (Fig. 1c). To identify the differentially expressed miRNAs, we employed selection criteria as *p* value less than or equal to 0.05 (significant) and linear fold change of at least ±1.5 or more. Thus, we obtained a total of 190 miRNAs (Fig. 1d and Supplementary Table S4) that were significant and differentially expressed. Surprisingly, only 7 miRNAs were found to be up-regulated and the remaining was downregulated. The top up-regulated miRNA was hsa-miR-668 with a fold change of 1.70. On the contrary, the fold change of the top downregulated miRNA was −7.62 for hsa-miR-923. The significantly differentially expressed miRNAs constituted 22.45% of the total miRNAs. The downregulated miRNAs constituted 21.63% of the total miRNAs, however, making it 96.31% of the total miRNAs which were significantly different in their expression in PTSD as compared to control.

### mRNA functional enrichment reveals dysregulation in immune system pathways in PTSD

**Figure 2:**
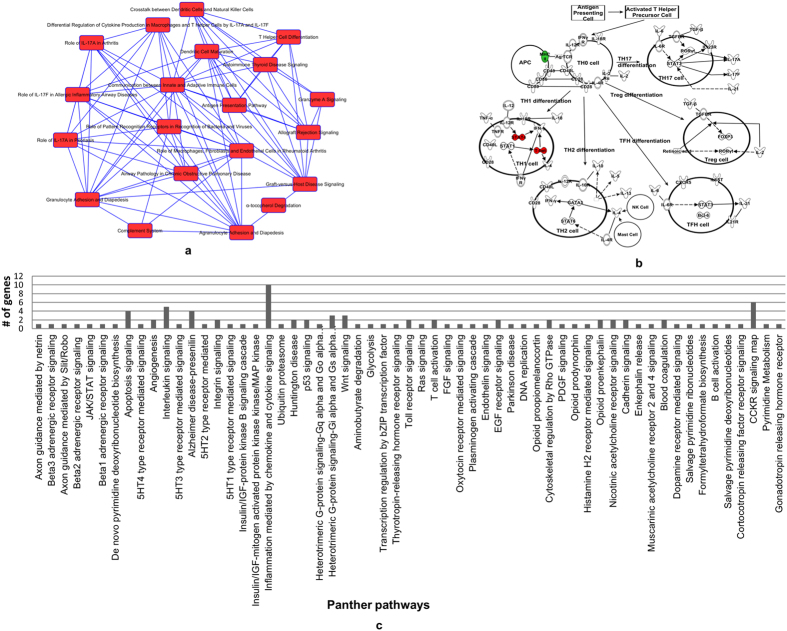
Expression of immune system related pathway genes are altered in PTSD. **(a)** The top 20 canonical pathways selected for finding the genes common in more than one canonical pathway (overlap). Many of the differentially expressed genes are present in multiple pathways related to immune system biology. Table 2 has the list of genes from our dataset that are present in all the canonical pathways in the list. **(b)** T helper cell differentiation canonical pathway with genes differentially expressed in PTSD. Red and green colors indicate up- and downregulated genes, respectively, in PTSD. The pathway was generated by analyzing all the differentially expressed genes in IPA. **(c)** The differentially expressed genes were analyzed on Panther pathways analysis tool. The Panther pathway with highest number of genes (10) from the dataset was “inflammation mediated by chemokine and cytokine signaling pathway” (P00031).

We first performed functional enrichment in IPA to identify canonical pathways with the 326 genes that indicated significantly differential expression. Majority of the pathways were related to immune system functioning/biology. To that end, we selected the top 20 canonical pathways for further analysis (Fig. 2a) of which we found Th cell differentiation pathway as the one that was most relevant to our studies (Fig. 2b). In this particular pathway, STAT4, TBX21 and HLA-DQA1 were differentially expressed. STAT4 and TBX21 were up-regulated and HLA-DQA1 was downregulated based on RNA-Seq analysis. The canonical pathways were then categorized on the basis of number of genes present from our dataset. The top canonical pathway revealed was ‘agranulocyte adhesion and diapedesis’ in which 16 of the genes from our dataset were present (Table 2, and Supplementary Fig. S1). Of the 16 genes, 4 were up- and 12 were downregulated. The up-regulated genes included CXCL2, CXCL3, CCL4 and CCL5. To give a clearer picture of the up- or downregulated genes in a specific canonical pathway, the list of genes and the top 20 canonical pathways are provided in Table 2. Thereafter, we used the differentially expressed genes for functional enrichment and gene ontology analysis by analyzing them in Panther pathways and DAVID as well. Based on the Panther pathway analysis (Fig. 2c), ‘inflammation mediated by chemokine and cytokine pathway (P00031)’ was the top pathway with 10 genes present from our dataset. Gene ontology analysis by DAVID revealed that the most significant (lowest *p* value, 3.80E–07) ontology was ‘immune system process’ with 40 genes from our dataset (Table 3).

### Expression pairing and other miRNA target analysis reveals differentially expressed miRNAs that target genes related to immune system in PTSD

**Figure 3:**
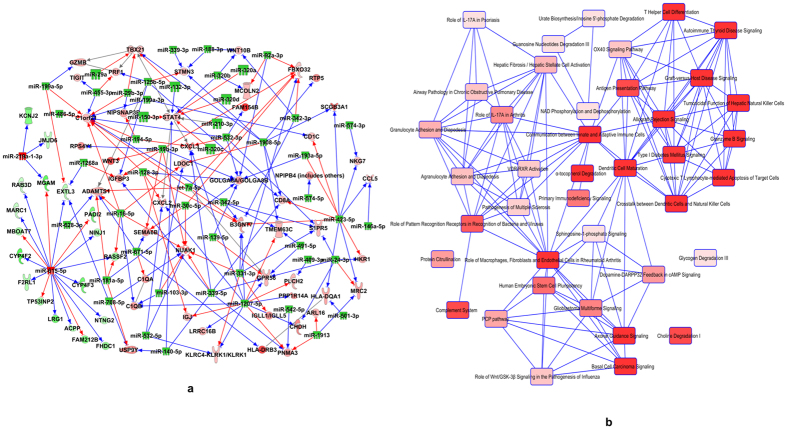
Expression pairing of the differentially expressed genes and miRNAs in PTSD. On IPA, both the miRNA microarray and RNA-Seq datasets were uploaded for Target Filter and performed expression pairing. **(a)** Expression paired molecules were used to generate a gene-miRNA interactive network (Green molecules indicate down- and red indicate up-regulation. Solid lines indicate direct interaction between a miRNA and a gene; green- experimentally proven; brown- highly predicted; and blue- moderately predicted interaction). (**b)** After expression pairing, the resulting genes that were inversely expressed with respect to miRNA expression were extracted (64 molecules) and uploaded in IPA for Core analysis. The figure shows top 40 overlapping canonical pathways obtained for genes after expression pairing.

We paired the expression of all the differentially expressed miRNAs (190) and genes (326) by using the IPA Expression pairing tool. From the total 190 differentially expressed miRNAs in the dataset, 44 (23.16%) miRNAs were identified to have targets in the RNA-Seq dataset during the time of analysis in IPA. Of the 326 differentially expressed genes, >70 (21.48%) were identified to be targets of the 44 paired miRNAs (Supplementary Fig. S2). A list of the paired genes and miRNAs is provided in supplementary files (Supplementary Table S5) and Fig. 3a shows the miRNAs and the target genes in the IPA-generated interactive network. Functional enrichment in IPA of the genes paired with the 44 miRNAs revealed that most of the canonical pathways were related to the immune system biology (Fig. 3b), similar to that observed in the first analysis (Fig. 2a). It is also worth mentioning that Th cell differentiation pathway (−log p value = 2.86E00) was one of the top canonical pathway obtained after expression pairing. Table 4 provides the list of the genes in all the top pathways shown in Fig. 3b.

As mentioned earlier, because all miRNAs or genes are not covered by one single database or tool, we wanted to see whether there are genes and miRNAs in our dataset that were not picked during Expression pairing in IPA, but are predicted to be target(s) of miRNAs in our dataset. Based on the miRNA target gene information available on www.targetscan.org website, we could identify many more miRNAs (78, list not exhaustive) that target the genes (top 10 differentially expressed) from our dataset. Table 5 provides names of the top ten differentially expressed genes from our dataset based on the highest fold change values and miRNAs with the highest relevancy scores as provided in www.targetscan.org.

### Altered DNA methylation pattern is evident during PTSD and correlates to differential gene expression

**Figure 4:**
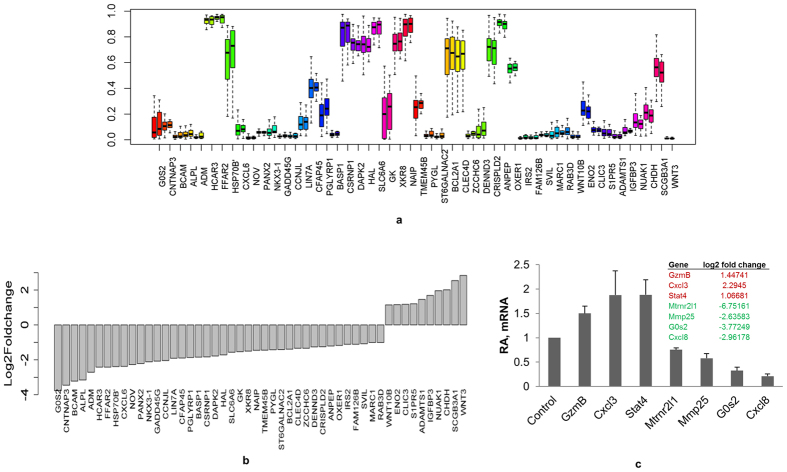
DNA methylation level has a trend that corroborates gene expression. There is a clear trend showing higher DNA methylation and lowered mRNA levels and vice-versa for the corresponding gene. **(a)** DNA methylation levels of the select genes presented as box plot. On x-axis, the names of gene are provided and y-axis provides the average β- values of DNA methylation. The two bars corresponding to each gene represent the DNA methylation level for control followed by PTSD patient in a left to right direction. **(b)** Transcript levels (y-axis: log 2 fold change values) of genes, after RNA-Seq analysis, listed in Fig. 4a. (c) Real time PCR validation of differentially expressed genes. To validate the RNA-Seq results, qRT-PCR was performed for seven representative genes with cDNA prepared from total RNA obtained from PBMCs of 24 control and 24 PTSD patients. The values are relative abundance (RA) values after qRT-PCR. The table inside the figure provides log 2 fold change values of the respective genes after RNA-Seq analysis. The error bars indicate standard error.

We compared the level of DNA methylation at the CpG sites of differentially expressed genes (326) present in our dataset. Based on the Illumina CpG site designations, only 138 genes from our dataset had true CpG sites. As per our set criteria used for this analysis, in the PTSD group, 40 (12.27%) genes had higher DNA methylation average β-values at their corresponding CpG sites when compared to that of controls (Supplementary Table S6) and these 40 genes had lower expression values as per RNA-Seq analysis. On the other hand, only 12 (3.68%) genes had decreased DNA methylation average β-values in PTSD (Supplementary Table S6), and the expression level of those genes was higher in PTSD group. In table 6, only the top 10 up- and downregulated genes with their DNA methylation levels are shown. The DNA methylation values were not significant as per Student’s *t-* test or Wilcoxon test. However, in all the genes listed in this study, there was a gradual trend of DNA methylation values supporting the expression levels of the genes (Fig. 4a provides DNA methylation levels of the genes as box plot; 4b provides fold change values of the genes, following RNA-Seq analysis, listed in Fig. 4a, and Table 6 provides DNA methylation levels of the top 20 genes).

### qRT-PCR confirmation of RNA-seq results

To validate our RNA-Seq data, we selected seven genes (*MTRNR2L1*, *MMP25*, *CXCL8*, *G0S2*, *GZMB*, *CXCL3 and STAT4*) as representative for qRT-PCR analysis. *MTRNR2L1*, *MMP25*, *CXCL8* and *G0S2* were shown to be significantly downregulated and *GZMB*, *CXCL3 and STAT4* significantly up-regulated by RNA-Seq analysis. The entire above mentioned gene expression matched with that of RNA-Seq results as per qRT-PCR data (Fig. 4c).

## Discussion

The prevalence of PTSD is high among war veterans and in the general public who experience traumatic events. However, the nature of changes occurring in PTSD, at the molecular level, in the PBMCs, is largely unclear. In the current study, we were therefore interested in exploring the global differences in the gene expression during active PTSD in PBMCs and, most importantly, correlate the difference with any altered epigenetic marks. Consequently, we first obtained global RNA expression pattern in the PBMCs obtained from war veterans diagnosed positive for PTSD during the time of sample collection and identified differentially expressed genes and the related immune system canonical pathways. A novel approach used in the present study is the strategy to combine RNA-Seq data with miRNA array data to simultaneously identify differentially expressed genes and their regulators at the global level. This approach provided broader information on the differentially expressed genes and their regulators by eliminating the limiting factors associated with single gene studies. Thus, we could correlate the differential expression of the target genes with the differential expression pattern of relevant miRNAs. To our knowledge, though preliminarily, this is the first time differentially expressed gene networks specific for the immune system are shown to correlate with global alteration of miRNA expression in the PBMCs of PTSD patients. Furthermore, we correlated the expression of several differentially expressed genes with altered DNA methylations at the corresponding CpG sites of the promoter of the respective genes.

Functional enrichment of the differentially expressed genes indicated probable alteration of Th cell differentiation pathway. This pathway was one of the major pathways with three genes (STAT4, TBX21, and HLA-DQA1) present from the significant set of genes. STAT4 and TBX21 have crucial roles in regulating T cell functions. For example, TBX21 is the main transcription factor for the expression of interferon gamma, a pro-inflammatory gene and already reported by our group (Bam *et al.*^7^) to be elevated in PTSD. This observation is important because the fate of Th cells decides the outcome of immune cell functions whether to be pro- or anti-inflammatory in nature. It also further supports the report on differential expression of T cell produced pro-inflammatory cytokine(s) in PTSD^6^. Therefore, we conclude that an alteration in the T cell biology is possibly one of the root causes for the underlying inflammation seen during PTSD. Altogether, our observation corroborates well with previous PTSD reports employing RNA-Seq technique with RNA obtained from peripheral blood leukocytes^8^. The authors reported that several genes involved in the innate immune system network were differentially expressed in PTSD. Similarly, in the present study, the top canonical pathways with differentially expressed genes were from the innate immune system in addition to some disease specific pathways. For example, the top canonical pathway with the largest number of differentially expressed genes from our dataset was ‘agranulocyte/granulocyte adhesion and diapedesis’. This pathway describes the stages involved in the movement or migration of leukocytes out of the circulatory system to the site of tissue damage or infection during an inflammatory response^32^,^33^,^34^. Chemotactic molecules (chemokines) like those secreted by monocytes, macrophages and other immune cells play an important role during this process^35^,^36^. Function of chemokines is mainly to bring about migration (homing) of leukocytes in the respective sites during homeostasis and inflammatory processes^37^. Other functions of chemokines are seen during different processes like maturation, activation and differentiation for different types of leukocytes^38^,^39^,^40^. We observed that expression of many of the chemokines and their receptors (CCL4, CCL5, CXCL1, CXCL2, CXCL3, CXCL6, CXCL8, CXCR1, and CXCR2) were altered in PTSD patients. Chemokines like CCL4, CCL5, CXCL1, CXCL2, CXCL3, CXCL6 and CXCL8 are considered to be pro-inflammatory in their function^41^. Another example of a canonical pathway with several differentially expressed genes from our dataset was “dendritic cell maturation”, which plays a critical role in antigen processing and presentation.

Micro-RNAs are critical regulators of gene expression and their interaction with target mRNAs can lead to destabilization of the mRNA in most (>90%) of the cases in mammals^23^. Expression of genes of the immune system is also known to be controlled by these small RNA molecules^42^. Interestingly, we found that several up-regulated genes in PTSD were either known or predicted to be a target of the many downregulated miRNAs from our dataset. For example, hsa-miR-125a and hsa-miR-193a-5p, which target Interferon gamma (*IFNG*)^6^ and Interleukin-12B (*IL12B*)^7^ respectively, were downregulated in PTSD as reported from our group previously. Both these genes are pro-inflammatory in their functioning^43^,^44^. As another example, we observed that *TBX-21* and *STAT4,* the key genes in the Th cell differentiation pathway, were upregulated in PTSD. TBX-21 is one of the main transcription factors involved in the differentiation and functioning of Th1 cells and also plays an important role in the functioning of other cells of the immune system^45^,^46^. *STAT4* is induced in response to signaling via the IL-12 pathway, leading to induction of *IFN* in Th1 type CD4+ cells^47^,^48^. There were several downregulated miRNAs from our dataset that were predicted to target *TBX-21* and *STAT4*. These observations suggested that several of the up-regulated genes in the PBMCs of PTSD patients could be resulting from the decreased presence of miRNAs, as a result of their downregulation, that target the genes post transcription. Further analysis of the miRNA dataset indicated that, up-regulated chemokines *CXCL2*, *CXCL3*, *CCL4* and *CCL5* are targets of numerous downregulated miRNAs from our dataset. Altogether, these observations suggest that there is a breakdown in the miRNA-mediated gene regulation in the PBMCs during PTSD and strikingly, it includes several of the up-regulated genes having pro-inflammatory properties. In contrast to the above observations, we found only 7 upregulated miRNAs and very few genes from our dataset were shown to be their targets. Moreover, these genes were not present in the top canonical pathways from our analysis.

We observed that a higher percentage (80.36%) of the differentially expressed genes in PTSD patients were downregulated. Correlating with the downregulated gene expression, there was a gradual but clear trend of higher DNA methylation level at CpG sites. Higher DNA methylation in the promoter region of a gene correlates with lower transcription of the gene and vice versa^27^,^28^,^29^. For example, our group has shown that *IL-12* transcription is increased in PTSD which correlated with lower DNA methylation at its promoter region^7^. Thus, it is possible that the genes in the PBMCs of PTSD patients are differentially expressed, at least in part, because of an altered DNA methylation. As another example, *CSRNP1* (aka *AXUD1*), a tumor suppressor, is one of the significantly downregulated genes in PTSD and it has higher level of DNA methylation in PTSD (82.08%) than control (79.09%). Downregulation of CSRNP1 correlates with progression of cancer^49^. This property can be extrapolated to reason that CSRNP1 downregulation can lead to higher cellular proliferation. Previously, our group has reported that CD4+ T cell, CD8+ T cell and B cell numbers are higher in PTSD patients^6^, and this is an indication that the proliferation of these cells is higher during PTSD. Thus, it is possible that the higher cell numbers of lymphocyte subsets seen in PTSD may result from lower CSRNP1 expression which probably is because of the higher DNA methylation at its promoter region. Furthermore, we found that *STAT4* is among the significantly up-regulated genes and has correspondingly lower DNA methylation trend at the CpG island. *STAT4*, induced by IL-12, leads to activation and proliferation of Th1 type CD4+ cells, which correlates well with our observation of increased T cells and alteration in the Th cell differentiation pathway in PTSD^6^. Thus, we hypothesize that the DNA methylation of immune system genes is altered during PTSD resulting in differential expression of a section of the genes.

In summary, the present work has identified differentially expressed genes and miRNAs and the related canonical immune system pathways in the PBMCs of PTSD inflicted war veterans. Furthermore, we provide evidence that many genes have altered DNA methylation at their CpG islands and the expression of the associated genes inversely correlate in PTSD patients. Taken together, the present and previous reports from our lab, and from other research groups, clearly indicate that miRNAs and DNA methylation play a critical role in the modulation of the immune system, with a special emphasis on chronic inflammation seen in PTSD. Most importantly, our findings, although preliminary, open future directions for studies in a pathway specific manner and targeting specific gene regulators to develop novel management strategies and therapies to control the inflammatory response seen during PTSD in war return veterans and the general population.

## Subjects, Materials and Methods

### Patients

All procedures performed in studies involving human participants were approved by the University of South Carolina Institutional Review Board and experimental methods and protocols were carried out in accordance with the approved guidelines. Samples were collected after obtaining proper informed and written consent from every participant. PTSD patients were Veterans of either the 1991 Persian Gulf war, or of the recent Iraq or Afghanistan wars, recruited from William Jennings Bryan Dorn Veterans Medical Center, as described earlier^6^. All of the donors were first clinically assessed by professionals for PTSD. Participants were evaluated by the psychometric properties of the PTSD Checklist (PCL)^50^ and the PTSD diagnosis was validated by the Clinician Administered PTSD Scale^51^ and the Diagnostic and Statistical Manual of Mental Disorder (DSM-V)^4^. (Demographics of the PTSD patients included for the microarray and RNA-Seq analyses is provided in Table 1). PTSD patients with current alcohol and other substance abuse, undergoing immunosuppressive drug treatment or having immunosuppressive disease, were excluded. For normal controls, age-matched healthy volunteers, who did not have any symptoms of active infection or any history of immune compromise such as HIV, cancer, pregnancy or on chronic steroid therapy, were recruited.

### Sample collection and RNA isolation

Peripheral blood samples (10–20 ml) were collected in EDTA coated collection tubes and PBMCs were isolated using Ficoll-Paque (GE Healthcare, Uppsala, Sweden) within 1 h from sample collection. PBMC viability was determined by trypan-blue exclusion. Using a universal kit (AllPrep DNA/RNA/miRNA Universal Kit, Qiagen, Valencia, CA) recommended for simultaneous isolation of high quality DNA and total RNA including miRNAs, all the three entities were isolated from the same ~ten million PBMCs and immediately frozen at −80 °C until use. For the miRNA microarray analysis, we used 4 controls and 8 PTSD samples. In the RNA-Seq analysis we used 5 controls and 5 PTSD samples. Two PTSD and 4 control samples were common for microarray and RNA-Seq analysis. For the qRT-PCR validation experiments, we included all the controls and PTSD samples used for the microarray and RNA-Seq analysis, in addition to more samples collected later, thereby making the total number of samples for each group to 24. For the DNA methylation data analysis, we included all the individuals’ (76 control and 23 PTSD) result submitted in the public database by the authors^52^.

### RNA-sequencing (RNA-Seq)

For RNA-Seq, five controls and five PTSD patients were analyzed. RNA-Seq libraries were constructed using Illumina TruSeq RNA Sample Preparation kit. Briefly, total RNA was purified from PBMCs using the Qiagen RNA easy kit. The oligo-dT beads were added to 1 μg of total RNA to isolate mRNA. The purified mRNA was fragmented to 200–400 bases. The RNA fragments were then reverse transcribed into double stranded cDNA fragments. The DNA fragments were repaired to generate blunt ends using T4 DNA polymerase, Klenow polymerase and T4 polynucleotide kinase. After DNA fragments were purified using Qiagen PCR purification kit (Qiagen catalogue #28004), an “A” base was added to the 3′ end of the blunt DNA fragment by Klenow fragment. Sequencing adapters were ligated to the ends of DNA fragments using DNA ligase. The libraries were then amplified by limited PCR (15 cycles) using primers provided by the kit. The PCR products were then separated by 2% agarose gel electrophoresis and fragments with sizes ranging from 250 bp to 400 bp were excised and purified using the QIAquick Gel Extraction Kit (Qiagen catalogue #28704). The concentration and distribution of the library were determined by a NanoDrop spectrophotometer (Thermo Scientific, Wilmington, DE). The library was sequenced by Illumina HiSeq 2000 at Tufts University Genomic core facility. During analysis, we trimmed three nucleotides from the 5′ end. Raw sequencing reads (50 bp single-end) were mapped to human genome build hg19 using Tophat 2^53^. We used the default parameters (TopHat2) present in Galaxy for the mapping. The accepted hits were used for assembling transcripts and estimating their abundance using Cufflinks. The differentially expressed gene, promoter usage and splicing form were determined by Cuffdiff and Cuffcompare^54^. We selected genes keeping a cutoff value of 2 or more fold change plus a p value < 0.005. The heat maps and links were generated using Circos software^55^. The RNA-Seq data is now available in NCBI’s GEO database (Accession# GSE83601).

### Micro-RNA microarray

For the miRNA microarray, four controls and eight PTSD patients were included in the study. Microarray for the miRNAs was performed by Johns Hopkins Memorial Institute (Deep Sequencing and Microarray Core Facility), Baltimore. Total RNA, including mRNA, miRNA and other small RNA molecules, were isolated from PBMC samples as described above. Next, total RNA samples were used in the analysis of miRNA differential expression by miRNA array hybridization assay using the Affymetrix miRNA-v1 gene chip. Array data normalization and quality control was performed as described previously by Zhou *et al.*^56^. Linear fold-changes in miRNA up-regulation or down-regulation were calculated to compare the differences of all the miRNAs expressed between PTSD patients and controls. A linear fold-change of at least plus or minus 1.5 was used as a cut off value for the inclusion of a miRNA. Moreover, only the miRNAs which were significant on the basis of *p* value (less than or equal to 0.05) calculated using student’s *t* test, were included for the analysis. We call these miRNAs differentially expressed. We used two tailed Student’s *t* test to get the *p* values. The miRNA array data is now available in ArrayExpress (Accession# E-MTAB-4880).

### Data analysis tools and functional enrichment of the genes

We employed Ingenuity Pathway Analysis (IPA, http://www.ingenuity.com/, QIAGEN, CA), Panther^57^ and DAVID^58^,^59^ for functional analysis of our datasets. IPA has tools to define interactions between miRNAs and target genes. It also has tools to identify the canonical pathways in which a given set of genes are involved. We also took advantage of the IPA Expression pairing tool to identify miRNAs and target genes from our datasets that are known or predicted to interact. Panther is a bioinformatics-based pathway analysis tool which can be used for functional enrichment of a given set of genes. Similarly, DAVID is also a bioinformatics-based tool to perform functional annotation or gene ontology identification and other categorization of a given set of genes. Both Panther and DAVID cannot be used for miRNA-gene interactions however, they both have an exhaustive list of functional annotation data.

Functional enrichment of a set of genes can help to identify the biological pathways in which the genes in a dataset are involved and also other genes with which a certain gene interacts, thereby providing a more meaningful understanding of the data. Consequently, we performed functional enrichment analysis in IPA, Panther pathways and DAVID with the RNA-Seq data which included all the genes with a fold-change of 2 or more and *p* value less than or equal to 0.005 (we call these genes differentially expressed). The three bioinformatics tools are similar in that they search for evidence of enrichment of genes in particular list of genes. However, not all databases have complete information for all the genes and their interactions. Thus, a more complete analysis is obtained if the same dataset is analyzed using different tools.

### Expression pairing of miRNAs and genes

In this part of the analysis, the differentially expressed genes and the miRNAs were analyzed together to see whether there are gene(s) that are targets of the miRNAs from our dataset obtained from similar samples in the present study. This type of analysis can help to simultaneously identify the differentially expressed genes and their regulators (miRNAs, in this case) in the same cells. To do this, we used a tool called Expression pairing, available in the Ingenuity’s microRNA target filter analysis section. This tool is useful to determine the miRNA(s) and gene(s) that interact with each other from a given set of miRNAs and genes when provided simultaneously as input datasets. To perform expression pairing, both the miRNA array followed by the RNA-Seq dataset was analyzed in IPA simultaneously. The tool then paired miRNAs with the genes from the dataset based on Ingenuity’s knowledge base for miRNAs and their targets. The genes obtained after expression pairing are either known or predicted to be a target of miRNA(s) from the list. Consequently, we got a list of genes from the input dataset and also miRNAs that could be their possible regulator for the observed differential expression. Subsequently, the genes obtained after expression pairing were used for another round of functional enrichment analysis for which, only IPA was used. This functional enrichment was performed to obtain the pathways in which the genes targeted by the miRNAs are involved.

### Micro-RNA target gene digging

In our miRNA dataset, most of the miRNAs from PTSD patients were downregulated. Based on our experience, IPA did not cover all of the miRNAs and their targets. So, we performed miRNA-target search to identify additional differentially expressed miRNAs and genes in our dataset, which possibly could be targeted by the miRNAs. We used the publicly available website^60^, www.targetscan.org for performing this analysis. One at a time, the genes from our dataset were analyzed in www.targetscan.org. After obtaining the list of miRNAs that target the genes, we manually searched for the miRNAs that are present in our dataset. As we had a very long list of differentially expressed genes, we listed miRNAs for only the top up- or downregulated genes as per the RNA-Seq analysis.

### DNA methylation analysis

For identifying differences in the DNA methylation level in specific genes, we used the publicly available Gene Expression Omnibus (GEO) datasets (GSE21282)^52^ from NCBI’s website ( www.ncbi.nlm.nih.gov/). This dataset contains DNA methylation analysis results from 76 controls and 23 PTSD individuals (average age 45.8 years with varied trauma exposures and PTSD scores defined clinically). In this dataset, the DNA methylation of CpG sites was obtained from whole blood cells. The observations in the above mentioned dataset are different from ours in that our observations are collected from PBMCs rather than whole blood cells. However, using this dataset and our own earlier Methylated DNA immunoprecipitation (MeDIP) data (not provided), we could previously identify and report differential expression of *IL12* as a result of alteration in DNA methylation and other epigenetic mechanisms in PBMCs from PTSD patients^7^. We obtained the average β values for the true CpG sites around TSS of specific genes from the datasets. For our purpose, we included all the β values without using a cutoff value. Moreover, in a case where there was more than one CpG site, we report here the β values of only the CpG sites with higher average β values. We compared the DNA methylation levels for all the differentially expressed genes from our RNA-Seq dataset which had true CpG islands and listed in the Illumina’s probe list.

### Quantitative Real Time PCR validation of the RNA-Seq data

For validation purpose of the RNA-Seq data, we selected seven genes for analysis. Complementary DNA (cDNA) was prepared from 0.5 μg of total RNA using miScript RT II kit (Qiagen, Valencia, CA) in a 20 μl system and used 7 ng of the original amount for qRT-PCR. Quantitative RT-PCR in triplicate wells was performed in an Applied Biosystem ViiA^TM^ 7 Real-Time PCR system (Life Technologies, Carlsbad, CA) real time PCR instrument. As an internal control, 18S rRNA and GAPDH message was quantified along with the genes. The expression level of the genes was expressed as the relative abundance value to the internal control. For the validation of gene expression by employing qRT-PCR, we included 24 controls and 24 PTSD samples.

## Additional Information

**How to cite this article**: Bam, M. *et al.* Dysregulated immune system networks in war veterans with PTSD is an outcome of altered miRNA expression and DNA methylation. *Sci. Rep.* **6**, 31209; doi: 10.1038/srep31209 (2016).

## Supplementary Material

Supplementary Information

## Figures and Tables

**Table 1:** Demographics and clinical history of the PTSD patients included for the microarray and RNA-Seq analysis.

For miRNA microarray analysis			
Parameters	Control (n = 4)	Patient (n = 8)	P-value
Age	40.5 (4.8)	37.2 (5.3)	0.323
Race			
AA*	2 (0.167)	3 (0.250)	
CA**	2 (0.167)	4 (0.333)	
Hisp***	0 (0.000)	1 (0.083)	0.571
Depression score	17 (4.3)	30.4 (9.6)	0.008
Anxiety score	14.8 (12.2)	29 (11.6)	0.102
PTSD score	43.5 (2.1)	62.9 (12.1)	0.002
**For RNA-Seq analysis**			
**Parameters**	**Control** (**n = 5)**	**Patient** (**n = 5)**	**P-value**
Age	42.6 (6.3)	38.4 (8.8)	0.414
Race			
AA*	2 (0.2)	4 (0.4)	
CA**	3 (0.3)	1 (0.1)	0.221
Depression score	14.4 (6.9)	38.6 (11.5)	0.006
Anxiety score	11.8 (12.4)	41.2 (13.5)	0.007
PTSD score	42.6 (2.7)	70.8 (13)	0.007

The mean (standard deviation) was used for continuous variable and the number (proportion) was used for categorical variable. Based on *t* test and Kruskal Wallis test, the age and race were comparable. Gender is not listed since all participants were males. The values indicate mean and the values in parentheses for age, depression, anxiety and PTSD score indicates standard deviation. Depression, anxiety and PTSD scores were determined as per PCL, CAPS and DSM criteria^4^,^50^,^51^.

^*^African American; **Caucasian; ***Hispanic.

**Table 2:** The top 20 canonical pathways and the distribution of the differentially expressed genes obtained after analysis in IPA with the 326 genes.

Canonical pathways	No. of genes	Gene symbol
Agranulocyte Adhesion and Diapedesis	16	*MMP25, MYL4, **CCL5**, MMP9, MYH10, **CXCL2**, CXCL8, CXCL6, IL1RN, C5AR1, CXCR1, CXCR2, CLDN9, CXCL1, **CXCL3, CCL4***
Granulocyte Adhesion and Diapedesis	15	*MMP25, **CCL5**, MMP9, FPR2, **CXCL2**, CXCL8, CXCL6, IL1RN, C5AR1, CXCR2, CLDN9, CXCL1, **CXCL3, CCL4**, HRH2*
Dendritic Cell Maturation	12	***STAT4**, IL1RN, CREB5, **HLA-DQA1, HLA-DRB3**, HLA-DRB5, **PLCH2**,HLA-C, **CD1C**, COL18A1, HLA-DRA, FCGR3A/FCGR3B*
Role of Macrophages, Fibroblasts and Endothelial Cells in Rheumatoid Arthritis	12	***WNT3**, OSM, F2RL1, IL1RN, **CCL5**, CREB5, C5AR1, **PLCH2**, TLR6, CXCL8, **WNT10B**, FCGR3A/FCGR3B*
Communication between Innate and Adaptive Immune Cells	10	*IL1RN, CD8A,CCL5,**HLA-DRB3**,HLA-DRB5,HLA-C, TLR6, CCL4, HLA-DRA, CXCL8*
Graft-versus-Host Disease Signaling	7	*IL1RN, **HLA-DQA1**, HLA-DRB5, **PRF1**, HLA-C, HLA-DRA, **GZMB***
Allograft Rejection Signaling	7	***HLA-DQA1, HLA-DRB3**, HLA-DRB5, **PRF1**, HLA-C, HLA-DRA, **GZMB***
Role of Pattern Recognition Receptors in Recognition of Bacteria and Viruses	7	*OSM,**CCL5**, C5AR1, **C1QB**, TLR6, CXCL8,*
Autoimmune Thyroid Disease Signaling	6	***HLA-DQA1**, HLA-DRB5, **PRF1**, HLA-C, HLA-DRA,*
Role of IL-17A in Arthritis	6	*CXCL6, **CCL5**, PTGS2, CXCL1, **CXCL3**, CXCL8*
Crosstalk between Dendritic Cells and Natural Killer Cells	6	***HLA-DRB3**, HLA-DRB5, **PRF1**, HLA-C, **KLRC4-KLRK1/KLRK1**, HLA-DRA*
Complement System	5	*C5AR1, **C1QB**, C4BPA, CR1,*
Antigen Presentation Pathway	5	***HLA-DQA1, HLA-DRB3**, HLA-DRB5, HLA-C, HLA-DRA*
Role of IL-17F in Allergic Inflammatory Airway Diseases	5	*CXCL6, CREB5, CXCL1, **CCL4**, CXCL8*
OX40 Signaling Pathway	5	***HLA-DQA1, HLA-DRB3**, HLA-DRB5, HLA-C, HLA-DRA*
Role of IL-17A in Psoriasis	4	*CXCL6, CXCL1, CXCL3, CXCL8*
Airway Pathology in Chronic Obstructive Pulmonary Disease	3	*MMP9, **CXCL3**, CXCL8*
Granzyme A Signaling	3	*HIST1H1C, **GZMA, PRF1***
Differential Regulation of Cytokine Production in Macrophages and T Helper Cells by IL-17A and IL-17F	3	***CCL5**, CXCL1,*
α-tocopherol Degradation	2	*CYP4F3, CYP4F2*

The ranking is based on the number of genes present in a pathway from our dataset. (In the gene list, italicized bold are up-regulated and rest downregulated).

**Table 3:** The top gene ontologies obtained from DAVID after analysis with the 326 differentially expressed genes.

Term	Genes (#)	*p*-Value	Genes	Fold enrichment
Immune system process	40	3.80E-07	CXCL1, AQP9, MMP9, CXCL3, HLA-DRB3, CXCL2, PGLYRP1, KLRK1, CXCR2, CXCL6, TLR6, CCL5, CCL4, HLA-DMA, HRH2, RASGRP4, HLA-DRB5, CLEC4D, NFIL3, FCGR3B, CR1, POU2AF1, C5AR1, JARID2, GZMA, NCF1, NCF4, IL1RN, CD1C, HLA-C, C4BPA, HLA-DQA1, OSM, C1QA, C1QB, JMJD6, AHSP, TREML2, PTAFR, HLA-DRA	2.42
Locomotion	16	0.005325	CXCL1, CMTM2, C5AR1, S100P, CXCL3, CXCL2, CXCR1, CXCR2, CXCL6, FPR2, CCL5, CCL4, PROK2, GAB2, PTAFR, MYH10	2.24
Response to stimulus	70	0.053086	AQP9, PTGS2, F2RL1, PGLYRP1, CXCR1, CXCR2, TLR6, HLA-DMA, MMP25, DYSF, MAP1LC3A, CLEC4D, FAM129A, NFIL3, FCGR3B, POU2AF1, IRS2, C5AR1, GZMA, NCF1, NCF4, HLA-C, HLA-DQA1, EEPD1, OSM, C1QA, RETN, PROK2, C1QB, TNFAIP6, THBD, ADM, F5, GADD45G, CA4, PPP1R15B, PTAFR, KDM6B, HLA-DRA, ALPL, CXCL1, PRF1, HLA-DRB3, CXCL3, CXCL2, NINJ1, FPR2, CXCL6, CCL5, CCL4, TRIB1, MEFV, RASGRP4, HRH2, ENO2, HLA-DRB5, TAS2R40, COL18A1, MAFF, HIST1H2BC, CR1, CMTM2, S100P, IL1RN, CD1C, C4BPA, NFKBIL1, S100A12, ORM1, MYH10	1.21
Multi-organism process	18	0.058821	MAFF, PRF1, HIST1H2BC, PTGS2, PGLYRP1, ANPEP, HLA-C, TEAD3, CCL5, TLR6, CCL4, UBN1, S100A12, TRIB1, THBD, ADM, PI3, PTAFR	1.60
Developmental process	63	0.067362	IER3, STEAP4, HKR1, PDLIM7, PTGS2, TUBB2A, MMP9, TBX21, ANPEP, HLA-DMA, WNT3, S1PR5, FRAT2, IFRD1, PHC2, IRS2, WNT10B, STX3, STMN3, DHRS9, MXD1, OSM, C1QB, PROK2, RETN, THBD, ADM, GADD45G, AHSP, RPS4Y1, CA4, ADAMTS1, NAIP, ALPL, CXCL1, MYL4, PLXNC1, ABHD5, NINJ1, CCL5, CCL4, EPHB1, B3GNT5, LRG1, RASGRP4, CRISPLD2, PPL, TGM3, NKX3-1, HIP1, COL18A1, MAFF, NFE2, JARID2, NTNG2, MICALCL, ISL2, SEMA6B, JMJD6, SVIL, CSRNP1, IGFBP3, MYH10	1.21
Death	18	0.09132	PRF1, IER3, GZMA, BCL2A1, CXCR2, GZMB, GZMH, DAPK2, NFKBIL1, OSM, TNFRSF10C, JMJD6, CSRNP1, GADD45G, NAIP, NEK6, AATK, HIP1	1.50

The rankings are based on the *p*-values, starting with the lowest. All the names of the genes present in our dataset are provided.

**Table 4:** The top canonical pathways and the genes from our dataset after functional enrichment of the genes obtained from expression pairing in IPA.

Canonical pathways	↓Down	↑Up	−log(p-value)	Genes	miR:RNA-seq Target Molecules (total # of significant mRNAs in pathway from RNA-seq)
α-tocopherol Degradation	2/4 (50%)	0/4 (0%)	4.22E00	2	CYP4F3, CYP4F2
Allograft Rejection Signaling	0/73 (0%)	4/73 (5%)	4.06E00	4	HLA-DQA1, HLA-DRB3, PRF1, GZMB
Dendritic Cell Maturation	0/176 0%	5/176 (3%)	3.61E00	5	STAT4, HLA-DQA1, HLA-DRB3, PLCH2, CD1C
Graft-versus-Host Disease Signaling	0/46 (0%)	3/46 (7%)	3.37E00	3	HLA-DQA1, PRF1, GZMB
Autoimmune Thyroid Disease Signaling	0/47 (0%)	3/47 (6%)	3.34E00	3	HLA-DQA1, PRF1,GZMB
Granzyme B Signaling	0/16 (0%)	2/16 (13%)	2.93E00	2	PRF1, GZMB
T Helper Cell Differentiation	0/69 (0%)	3/69 (4%)	2.86E00	3	STAT4, HLA-DQA1, TBX21
Basal Cell Carcinoma Signaling	0/72 (0%)	3/72 (4%)	2.8E00	3	WNT3, HKR1, WNT10B
Axonal Guidance Signaling	0/432 (0%	6/432 (1%)	2.6E00	6	WNT3, SEMA6B, PLCH2, HKR1, ADAMTS1, WNT10B
Role of MΦ, Fibroblasts and Endothelial Cells in RA	1/297 (0%)	4/297 (1%)	2.59E00	5	WNT3, F2RL1, CCL5, PLCH2, WNT10B
Tumoricidal Function of Hepatic Natural Killer Cells	0/24 (0%)	2/24 (8%)	2.58E00	2	PRF1, GZMB
Communication between Innate and Adaptive Immune Cells	0/91 (0%)	3/91 (3%)	2.51E00	3	CD8A, CCL5, HLA-DRB3
Cytotoxic T Lymphocyte-mediated Apoptosis of Target Cells	0/32 (0%)	2/32 (6%)	2.33E00	2	PRF1, GZMB
Type I Diabetes Mellitus Signaling	0/108 0%	3/108 (3%)	2.3E00	3	HLA-DQA1, PRF1, GZMB
Antigen Presentation Pathway	0/37 (0%)	2/37 (5%)	2.21E00	2	HLA-DQA1, HLA-DRB3
Complement System	0/37 (0%)	2/37 (5%)	2.21E00	2	C1QB, C1QA
Choline Degradation I	0/2 (0%)	1/2 (50%)	2.2E00	1	CHDH
Role of Pattern Recognition Receptors in Recognition of Bacteria and Viruses	0/125 (0%)	3/125 (2%)	2.13E00	3	CCL5, C1QB, C1QA
Human Embryonic Stem Cell Pluripotency	0/133 (0%	3/133 (2%)	2.06E00	3	WNT3, WNT10B, S1PR5
Glioblastoma Multiforme Signaling	0/146 0%	3/146 (2%)	1.95E00	3	WNT3, PLCH2, WNT10B
Role of IL-17A in Arthritis	0/54 (0%)	2/54 (4%)	1.89E00	2	CCL5, CXCL3 (of 6)
Dopamine-DARPP32 Feedback in cAMP Signaling	1/161 (1%)	2/161 (1%)	1.83E00	3	KCNJ2, PPP1R14A, PLCH2
Protein Citrullination	1/5 (20%)	0/5 (0%)	1.8E00	1	PADI2
PCP pathway	0/63 (0%)	2/63 (3%)	1.76E00	2	WNT3, WNT10B
Granulocyte Adhesion and Diapedesis	0/177 (0%	3/177 (2%)	1.72E00	3	CCL5, CXCL3, CXCL2
Agranulocyte Adhesion and Diapedesis	0/189 (0%	3/189 (2%)	1.65E00	3	CCL5, CXCL3, CXCL2
OX40 Signaling Pathway	0/76 (0%)	2/76 (3%)	1.61E00	2	HLA-DQA1, HLA-DRB3

The rankings are based on the *p* values, starting with the lowest.

**Table 5:** List of the top 10 up- and downregulated genes and the miRNAs from our dataset which are predicted or known to interact based on http://www.targetscan.org analysis.

Target	log 2 fold change*	miRs
FAM154B	3.94	hsa-miRs-150-5p, -92a-1-5p, 15b-5p, -223-3p, -151-3p
WNT3	2.83	hsa-miRs-145-5p, -15b-5p, -149-3p, -23b-3p, -30c-2-3p, -30c-1-3p, -342-3p
SCGB3A1	2.54	hsa-miRs-423-5p, -663a, -625-5p, -30e-3p
CXCL3	2.3	hsa-mirs-425-5p, let-7c-3p, -532-3p, -584-5p, -1207-5p, -132-3p, -181-a-5p, -181b-5p, -181c-5p, -181d-5p, -150-5p, -194-5p
USP9Y	2	hsa-miRs-132-3p, -130b-3p, -130a-3p, -140-5p, -28-3p, -92b-3p, -92a-3p, -181a-5p, -181b-5p, -181c-5p, -181d-5p, -23a-3p
CHDH	2	hsa-miRs-455-3p, -342-5p, -1231, -140-3p, -28-5p, -29b-2-5p, -324-3p, -505-5p
NUAK1	1.96	hsa-miRs-455-3p, -28-5p, -107, -145-5p, -182-5p, -192-5p, -339-5p, -345-5p, -505-5p, -532-5p, -625-5p, -629-3p, -744-5p, -940
RPS4Y1	1.86	hsa-miRs-140-3p, -150-5p, -324-3p
PPP1R14A	1.83	hsa-miRs-1207-5p, let-7a-3p, -let-7b-3p, let-7f, -1228-5p
IGLL5	1.78	hsa-miRs-494-5p, -486-5p, --638, -143-3p, -193b-3p, -29b-1-5p, -331-3p, -486-5p

^*^log 2 fold change after RNA-Seq analysis.

**Table 6:** The top ten up- or downregulated genes from our dataset and the DNA methylation percentage of their corresponding CpG sites obtained from GSE21282 GEO datasets.

Gene id	Gene	log 2FC, RNA-Seq*	Control (%)	PTSD (%)
NM_015714	G0S2	−3.77249	10.7	11.5
NM_033655	CNTNAP3	−3.45161	10.8	11.0
NM_005581	BCAM	−3.22104	4.0	5.7
NM_000478	ALPL	−3.15304	4.6	8.0
NM_001124	ADM	−2.71289	2.9	4.6
NM_006018	HCAR3	−2.41909	87.8	89.4
NM_005306	FFAR2	−2.41673	89.7	93.1
NM_002155	HSP70B	−2.38861	62.4	65.9
NM_002993	CXCL6	−2.37093	8.3	11.1
NM_002514	NOV	−2.26268	2.5	3.0
NM_003394	WNT10B	1.15246	3.6	2.8
NM_001975	ENO2	1.1608	25.6	23.0
NM_004669	CLIC3	1.18195	7.9	7.1
NM_030760	S1PR5	1.20954	6.4	5.8
NM_006988	ADAMTS1	1.46005	3.6	2.7
NM_000598	IGFBP3	1.68741	8.7	7.9
NM_014840	NUAK1	1.96002	15.5	12.1
NM_018397	CHDH	2.00633	22.4	18.5
NM_052863	SCGB3A1	2.54427	56.1	52.2
NM_030753	WNT3	2.83619	3.3	2.1

^*^log2 fold change after RNA-Seq analysis.
