# Supplementary material for: Dysregulated immune system networks in war veterans with PTSD is an outcome of altered miRNA expression and DNA methylation
Source: Sci Rep. 2016 Aug 11;6:31209. doi: 10.1038/srep31209 (PMC4980621; doi:10.1038/srep31209)
Supplement: Supplementary Information [file srep31209-s1.pdf]

Dysregulated immune system networks in war veterans with PTSD is an outcome of altered miRNA expression and DNA methylation.

Marpe Bam[1,3,Ph.D], Xiaoming Yang[1,2,3,Ph.D], Elizabeth E. Zumbrun[1,Ph.D], Yin Zhong[1,BS], Juhua Zhou[1,4,Ph.D], Jay P. Ginsberg[2,Ph.D], Quinne Leyden[2,Psy.D], Jiajia Zhang[5], Prakash S. Nagarkatti[1,Ph.D] and Mitzi Nagarkatti[1,2,*,Ph.D]

[1]Department of Pathology, Microbiology and Immunology, University of South Carolina School of Medicine, Columbia, SC 29209, USA; [2]William Jennings Bryan Dorn Veterans Medical Center, 6439 Garners Ferry Road, Columbia, South Carolina 29209-1639; [4]Current Address: Institute for Tumor Immunology, Ludong University School of Life Sciences, Yantai, Shandong 264025, P.R. China; [5] Department of Epidemiology and Biostatistics, Arnold School of Public Health, University of South Carolina, Columbia, SC 29206, USA

[*]Mitzi Nagarkatti, 6439 Garners Ferry Road, Department of Pathology, Microbiology and Immunology, University of South Carolina School of Medicine, Columbia, SC 29209, United States. E-mail: mitzi.nagarkatti@uscmed.sc.edu; Phone: 803-216-3404; Fax: 803-216-3413

[3]These authors contributed equally to this work.

Supplementary Information

Supplementary Figure S1. Stacked bar chart showing the top 20 canonical pathways with the percentage of genes present from our dataset. For example, in the top pathways, there are 175 genes in total and ~10 percent of them are present in our dataset.

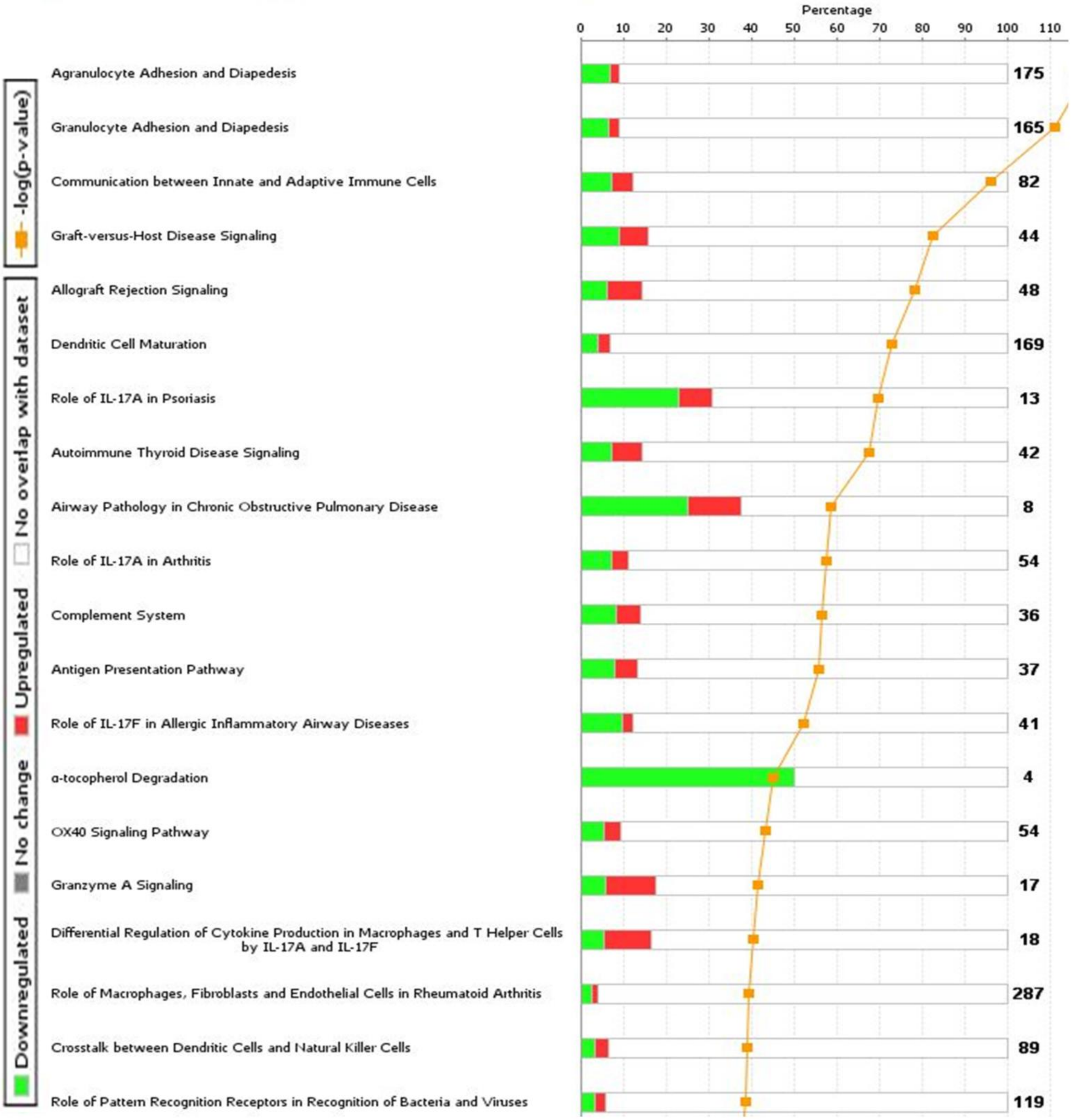

Supplementary Figure S2. Micro-RNA and gene interactions displayed as circos plot. RNA-seq was performed on five each control and PTSD samples and the $\log_2$ fold change of the average of the FPKM values obtained. The circos plot shows the $\log_2$ fold change of the expression level of the genes and their location on different chromosomes of *Homo sapiens*. The outer circle represents control and the inside for PTSD patients. The colored lines inside the circos plot depict miRNAs where each individual line represents one miRNA. The colored lines connect the chromosomal location of the miRNA and their target genes. Red line indicates up-regulated and green for downregulated miRNAs. The miRNAs were obtained after expression pairing with the RNA-Seq data.

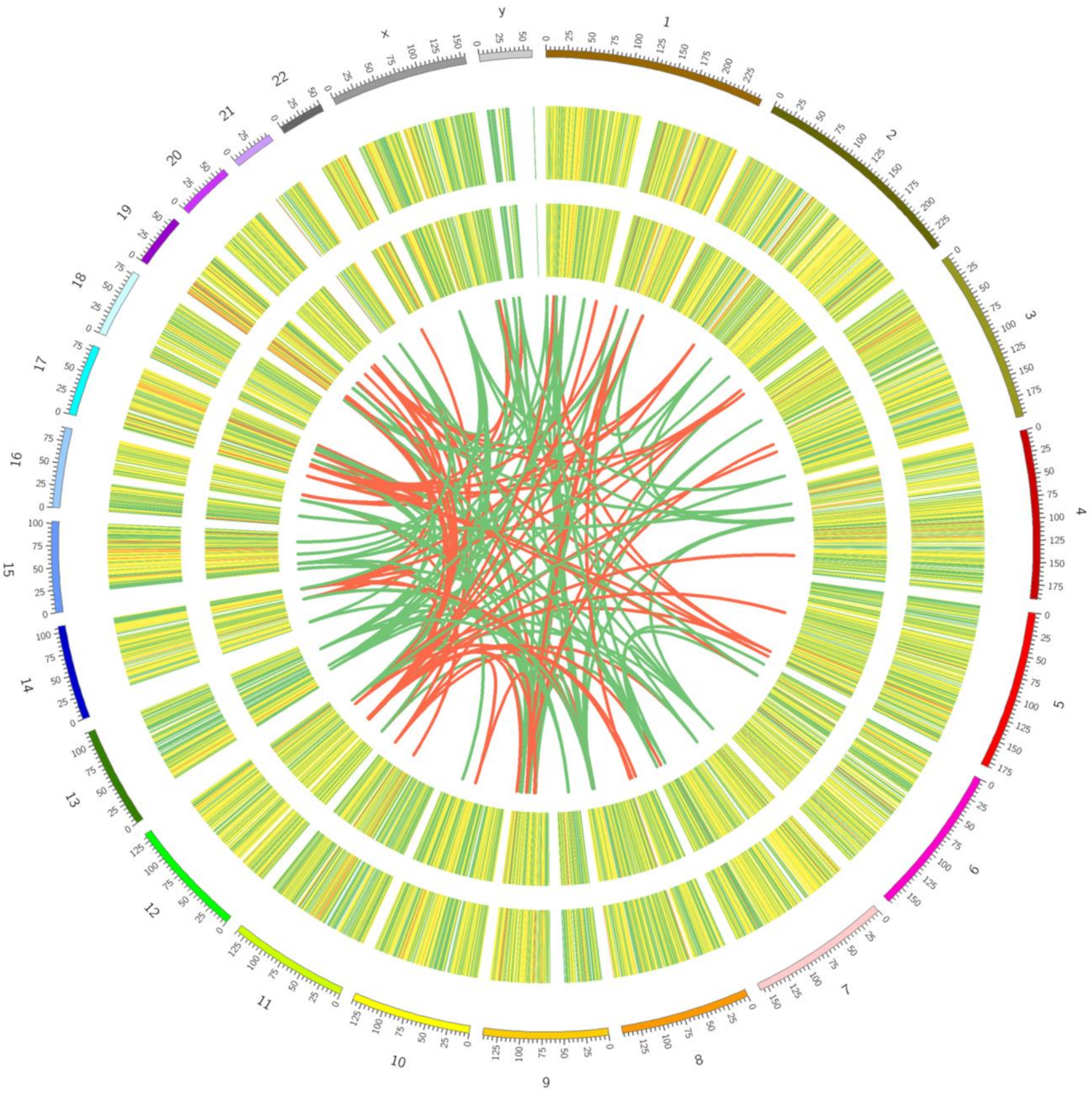

Supplementary Table S3: List of significantly altered genes in PTSD patients with p value <0.005 and log2 fold change of at least 1 unit.

**Gene id**	**log2(fold change)**
NM_001144963	∞ (infinity)
NM_003897	∞ (infinity)
NM_006120	∞ (infinity)
NM_032787	∞ (infinity)
NM_001008226	3.94796
NM_022555	3.08504
NM_030753	2.83619
NM_052863	2.54427
NM_002090	2.2945
NM_001144931	2.18939
NM_004654	2.0082
NM_018397	2.00633
NM_014840	1.96002
NM_173821	1.93352
NM_001008	1.86473
NM_033256	1.83417
NM_001178126	1.78344
NM_152353	1.77666
NM_001135865	1.70078
NM_000598	1.68741
NM_000491	1.6866
NM_015991	1.68385
NM_001023567	1.66771
NM_145236	1.56786
NM_032108	1.5555
NM_002122	1.54365
NM_002984	1.5327
NM_002089	1.52976
NM_006039	1.52444
NM_013364	1.46958
NM_006988	1.46005
NM_004131	1.44741
NM_020415	1.40461
NM_138360	1.39786
NM_016459	1.35587
NM_181786	1.33046
NM_173574	1.28499
NM_001077665	1.28039
NM_005041	1.27945
NM_020431	1.2778

NM_001040025	1.27629
NM_033423	1.27493
NM_012317	1.26908
NM_001145770	1.25437
NM_001303012	1.24512
NM_030760	1.20954
NM_030806	1.19672
NM_144646	1.19637
NM_004669	1.18195
NM_153259	1.17901
NM_001975	1.1608
NM_007360	1.15465
NM_003394	1.15246
NM_002985	1.14935
NM_013351	1.14426
NM_003970	1.12269
NM_173799	1.1102
NM_005601	1.08888
NM_015894	1.07848
NM_003151	1.06681
NM_006144	1.04488
NM_001765	1.01915
NM_006235	1.01571
NM_058229	1.00345
NM_004283	-1.00505
NM_022746	-1.00704
NM_030636	-1.01648
NM_207336	-1.01943
NM_015549	-1.04993
NM_018071	-1.05012
NM_001080497	-1.05324
NM_014643	-1.05607
NM_012154	-1.0637
NM_003174	-1.07427
NM_178815	-1.07435
NM_020775	-1.08718
NM_005319	-1.08883
NM_001282948	-1.09151
NM_032582	-1.1071
NM_173822	-1.10926
NM_003749	-1.11358
NM_032536	-1.12188
NM_016006	-1.12255
NM_032047	-1.12609
NM_004567	-1.12928
NM_006931	-1.14342

NM_015167	-1.14587
NM_170604	-1.14601
NM_004973	-1.14708
NM_173791	-1.15374
NM_018053	-1.1578
NM_001304	-1.17417
NM_148962	-1.17745
NM_024576	-1.18048
NM_001550	-1.18667
NM_030627	-1.18754
NM_023037	-1.18824
NM_001150	-1.2071
NM_022473	-1.21366
NM_001127208	-1.21585
NM_138774	-1.216
NM_014214	-1.21814
NM_014681	-1.22175
NM_005964	-1.22771
NM_004427	-1.23851
NM_001077446	-1.24249
NM_006068	-1.24397
NM_130445	-1.24522
NM_001164721	-1.24733
NM_031476	-1.25072
NM_004148	-1.25711
NM_000130	-1.25994
NM_005621	-1.26102
NM_014957	-1.26401
NM_020698	-1.26424
NM_001440	-1.26881
NM_001736	-1.26917
NM_001145841	-1.26998
NM_015652	-1.27046
NM_001098634	-1.27844
NM_014705	-1.28761
NM_000243	-1.29162
NM_032833	-1.29557
NM_030938	-1.30077
NM_001136023	-1.31609
NM_018340	-1.31792
NM_000265	-1.31878
NM_032898	-1.32018
NM_032867	-1.32376
NM_024807	-1.32474
NM_024617	-1.33553
NM_080387	-1.34041

NM_012198	-1.34266
NM_001785	-1.34843
NM_005242	-1.35807
NM_001134194	-1.35835
NM_001135726	-1.36603
NM_005502	-1.36766
NM_152680	-1.36958
NM_000361	-1.37013
NM_005338	-1.3752
NM_080491	-1.37706
NM_032412	-1.38116
NM_014397	-1.38165
NM_014465	-1.38406
NM_001080424	-1.38617
NM_020808	-1.39077
NM_004049	-1.39153
NM_001013627	-1.39533
NM_004177	-1.40273
NM_001174116	-1.40734
NM_006456	-1.41191
NM_000573	-1.412
NM_002863	-1.4257
NM_194255	-1.432
NM_000561	-1.43228
NM_145719	-1.44543
NM_138788	-1.44668
NM_001002814	-1.45221
NM_004536	-1.45422
NM_001079514	-1.45666
NM_024569	-1.46426
NM_001011666	-1.46846
NM_024298	-1.47451
NM_001272013	-1.48877
NM_031890	-1.48926
NM_021202	-1.48949
NM_003830	-1.49086
NM_001817	-1.50029
NM_001206880	-1.50141
NM_021083	-1.5015
NM_001005738	-1.50903
NM_000963	-1.52102
NM_198540	-1.52286
NM_032514	-1.52327
NM_005384	-1.52803
NM_000167	-1.53498
NM_006635	-1.53499

NM_001122770	-1.5355
NM_004441	-1.53607
NM_030759	-1.53734
NM_012083	-1.5448
NM_173842	-1.5511
NM_014737	-1.55809
NM_002357	-1.55969
NM_003043	-1.56557
NM_020982	-1.5677
NM_032283	-1.57011
NM_014844	-1.57672
NM_024081	-1.57966
NM_003530	-1.58063
NM_024607	-1.59013
NM_001069	-1.60656
NM_138799	-1.62584
NM_020530	-1.64337
NM_144669	-1.64898
NM_001007253	-1.65293
NM_014991	-1.65963
NM_000631	-1.67144
NM_016332	-1.67216
NM_005451	-1.67304
NM_018485	-1.6736
NM_145805	-1.67932
NM_001145678	-1.68198
NM_001131055	-1.68705
NM_002638	-1.68733
NM_001301104	-1.68909
NM_007115	-1.69977
NM_012413	-1.71433
NM_001286646	-1.71891
NM_002108	-1.71994
NM_005746	-1.72207
NM_012323	-1.7221
NM_001080975	-1.73976
NM_033393	-1.75358
NM_005761	-1.77812
NM_022481	-1.77862
NM_014326	-1.77918
NM_203447	-1.78357
NM_001207030	-1.82517
NM_052966	-1.82872
NM_033027	-1.83734
NM_006317	-1.84228
NM_199204	-1.86234

NM_013450	-1.86509
NM_198578	-1.86856
NM_005091	-1.87153
NM_032571	-1.8899
NM_000804	-1.89019
NM_012337	-1.89692
NM_004566	-1.89998
NM_004664	-1.90634
NM_024940	-1.91588
NM_001134337	-1.91748
NM_003059	-1.9269
NM_003526	-1.95023
NM_025195	-1.97313
NM_025144	-1.98928
NM_001815	-1.99143
NM_001042388	-2.00888
NM_024565	-2.0358
NM_000607	-2.05624
NM_019099	-2.06305
NM_006705	-2.06904
NM_006167	-2.11232
NM_170776	-2.11943
NM_016612	-2.12327
NM_000891	-2.12374
NM_002476	-2.1261
NM_001205315	-2.12789
NM_001291703	-2.13235
NM_001126128	-2.14825
NM_002125	-2.16189
NM_001013706	-2.17365
NM_001143769	-2.18116
NM_018050	-2.19678
NM_152891	-2.1969
NM_005139	-2.19989
NM_138782	-2.21169
NM_052839	-2.21281
NM_001276	-2.22245
NM_001003938	-2.22461
NM_004997	-2.23149
NM_019111	-2.23853
NM_000634	-2.24542
NM_178554	-2.24564
NM_198153	-2.25377
NM_032564	-2.2616
NM_002514	-2.26268
NM_020980	-2.26772

NM_007365	-2.29123
NM_001511	-2.30606
NM_020741	-2.31643
NM_178500	-2.32955
NM_002993	-2.37093
NM_002155	-2.38861
NM_016633	-2.38882
NM_015431	-2.40812
NM_052972	-2.41457
NM_005306	-2.41673
NM_024911	-2.41874
NM_006018	-2.41909
NM_001143804	-2.43018
NM_176882	-2.43756
NM_177551	-2.45439
NM_020406	-2.47961
NM_000570	-2.47966
NM_175710	-2.47979
NM_003841	-2.48047
NM_000896	-2.48078
NM_001282742	-2.48132
NM_019065	-2.50699
NM_001199317	-2.51979
NM_005980	-2.53461
NM_001557	-2.54225
NM_001159710	-2.57339
NM_001291832	-2.59714
NM_022468	-2.63583
NM_004668	-2.69177
NM_003214	-2.69864
NM_001124	-2.71289
NM_001293626	-2.73667
NM_000902	-2.76579
NM_004665	-2.77106
NM_004994	-2.7854
NM_002705	-2.88847
NM_001243042	-2.90174
NM_003245	-2.92565
NM_015297	-2.93336
NM_001082	-2.94352
NM_000584	-2.96178
NM_001080395	-2.97159
NM_001130981	-2.99527
NM_001130980	-3.08661
NM_001172631	-3.11641
NM_001145248	-3.1459

NM_000478	-3.15304
NM_005581	-3.22104
NM_000715	-3.26569
NM_000717	-3.37721
NM_033655	-3.45161
NM_001010987	-3.47877
NM_015515	-3.56923
NM_001039570	-3.65754
NM_015714	-3.77249
NM_001190452	-6.75161
NR_037194	-3.69867
NR_110907	-3.33775
NR_026817	-3.0604
NR_103549	-2.76247
NR_037142	-2.59941
NR_126161	-2.55986
NR_028324	-2.38937
NR_027072	-2.25576
NR_103548	-2.17617
NR_027256	-2.11981
NR_047572	-1.97086
NR_037867	-1.93293
NR_126168	-1.90112
NR_002189	-1.8776
NR_109769	-1.77106
NR_036534	-1.70274
NR_103791	-1.54662
NR_103718	-1.45414
NR_003187	-1.37966
NR_028502	-1.32709
NR_120420	-1.31823
NR_003186	-1.31434
NR_003505	-1.2379
NR_039983	-1.16287
NR_104116	-1.06893
NR_040085	1.06824
NR_027353	1.12582
NR_033841	1.17126
NR_024433	1.29705
NR_027293	1.35455
NR_024458	1.44115
NR_024011	1.5203
NR_036466	1.6418
NR_030732	1.77503
NR_026958	1.89413
NR_028062	2.18775

NR_038929	2.18956
NR_038402	2.24131
NR_047651	2.57877
NR_001434	3.52181

Supplementary Table S4: List of significantly altered miRNAs in PTSD with p value <0.05 and linear fold change of at least 1.5 units.

miRNAs	linear fold change
hsa-miR-668	1.704687008
hsa-miR-219-1-3p	1.624110733
hsa-miR-637	1.603837943
hsa-miR-518f-star	1.578372937
hsa-miR-570	1.563301993
hsa-miR-615-5p	1.539442786
hsa-miR-519a-star	1.515065588
hsa-miR-671-5p	-1.513276379
hsa-miR-186-star	-1.533234376
hsa-miR-802	-1.536867059
hsa-miR-191-star	-1.538949883
hsa-let-7b-star	-1.544565846
hsa-miR-938	-1.549924698
hsa-miR-21-star	-1.562938624
hsa-miR-140-5p	-1.584066608
hsa-miR-193b	-1.584079372
hsa-miR-182	-1.602199447
hsa-miR-933	-1.603837943
hsa-miR-301a	-1.604230837
hsa-miR-92a-1-star	-1.610969718
hsa-miR-188-3p	-1.612124317
hsa-miR-769-5p	-1.619629275
hsa-miR-331-3p	-1.628403243
hsa-miR-29b-1-star	-1.631073308
hsa-miR-1271	-1.63866009
hsa-miR-128	-1.648492416
hsa-miR-1231	-1.655907976
hsa-miR-1224-5p	-1.658029011
hsa-miR-30e-star	-1.664775052
hsa-miR-338-5p	-1.670223594
hsa-miR-139-5p	-1.693009419
hsa-miR-29b-2-star	-1.699438171
hsa-miR-331-5p	-1.703534748
hsa-miR-500-star	-1.706636579
hsa-miR-22	-1.725784709
hsa-miR-210	-1.726941395
hsa-miR-532-5p	-1.741030377
hsa-miR-92b	-1.751464352
hsa-miR-98	-1.77113094
hsa-miR-542-5p	-1.783131839
hsa-miR-30e	-1.789848063

hsa-miR-152	-1.791244159
hsa-miR-628-3p	-1.791556913
hsa-miR-29b	-1.800038015
hsa-miR-324-3p	-1.808019672
hsa-miR-24-2-star	-1.809127391
hsa-miR-491-5p	-1.814149374
hsa-miR-664-star	-1.816455944
hsa-miR-424-star	-1.828267124
hsa-miR-487b	-1.839408293
hsa-miR-502-3p	-1.842120102
hsa-miR-877	-1.843238173
hsa-miR-18a	-1.856575197
hsa-miR-181a-star	-1.866643763
hsa-miR-409-3p	-1.9283917
hsa-miR-26b	-1.947325427
hsa-miR-1228	-1.953007853
hsa-miR-503	-1.963493062
hsa-miR-940	-1.967491291
hsa-miR-194	-1.992670263
hsa-miR-378	-1.993033555
hsa-miR-324-5p	-1.99456498
hsa-miR-486-5p	-2.005740724
hsa-miR-106b	-2.015959806
hsa-miR-21	-2.020591768
hsa-miR-29a	-2.026130537
hsa-miR-1275	-2.027399815
hsa-miR-20a	-2.043663261
hsa-miR-28-5p	-2.049745083
hsa-miR-422a	-2.058926209
hsa-miR-339-3p	-2.059662922
hsa-miR-93	-2.05989118
hsa-miR-150-star	-2.061832664
hsa-miR-17-star	-2.066139802
hsa-miR-20b	-2.072629327
hsa-miR-363	-2.082007297
hsa-miR-494	-2.083476929
hsa-miR-99b	-2.113739554
hsa-miR-941	-2.116585657
hsa-miR-432	-2.119353693
hsa-miR-532-3p	-2.135527535
hsa-miR-15a	-2.142230916
hsa-miR-132	-2.152585997
hsa-miR-146a	-2.153401375
hsa-miR-193a-5p	-2.170490525
hsa-miR-16	-2.216802127
hsa-miR-339-5p	-2.224297675

hsa-miR-425-star	-2.228973941
hsa-miR-192	-2.231602194
hsa-miR-106a	-2.234719489
hsa-miR-1301	-2.237813218
hsa-miR-421	-2.240169079
hsa-miR-31	-2.241966739
hsa-miR-629-star	-2.26034717
hsa-miR-629	-2.266320461
hsa-miR-17	-2.279413244
hsa-miR-146b-5p	-2.282075069
hsa-miR-1307	-2.285837546
hsa-miR-24	-2.290785696
hsa-miR-1246	-2.305254447
hsa-miR-103	-2.307375011
hsa-miR-125b	-2.330878009
hsa-miR-744	-2.347473709
hsa-miR-27b	-2.347793261
hsa-miR-423-5p	-2.353091629
hsa-miR-766	-2.35338605
hsa-miR-181d	-2.365123059
hsa-miR-574-5p	-2.365947195
hsa-miR-99a	-2.389082419
hsa-miR-222	-2.407223966
hsa-miR-28-3p	-2.431094544
hsa-miR-505-star	-2.434437142
hsa-miR-185	-2.435432925
hsa-miR-1825	-2.442791322
hsa-miR-768-3p	-2.443767655
hsa-miR-423-3p	-2.44491151
hsa-miR-455-3p	-2.448347775
hsa-miR-30d	-2.455219697
hsa-miR-92b-star	-2.460492937
hsa-let-7i	-2.462854672
hsa-miR-106b-star	-2.473040515
hsa-miR-181a-2-star	-2.478217825
hsa-miR-345	-2.481643276
hsa-miR-181b	-2.505133305
hsa-let-7g	-2.509784728
hsa-miR-330-3p	-2.514597076
hsa-miR-30b	-2.524190204
hsa-miR-19b	-2.538700359
hsa-miR-130b	-2.53992276
hsa-miR-200c	-2.577618047
hsa-miR-652	-2.584204515
hsa-miR-93-star	-2.586868557
hsa-miR-107	-2.631118544

hsa-miR-574-3p	-2.641312728
hsa-miR-125a-5p	-2.644358906
hsa-miR-425	-2.668503447
hsa-miR-143	-2.679949843
hsa-miR-625	-2.714145617
hsa-miR-768-5p	-2.726943861
hsa-miR-1280	-2.729666596
hsa-miR-23a	-2.761756739
hsa-miR-140-3p	-2.767785231
hsa-miR-663	-2.774713134
hsa-miR-181c	-2.807895024
hsa-miR-151-3p	-2.83430055
hsa-miR-342-3p	-2.847932593
hsa-miR-320a	-2.886611594
hsa-miR-342-5p	-2.887232424
hsa-miR-26a	-2.888701735
hsa-miR-191	-2.909399174
hsa-miR-320b	-2.913674568
hsa-miR-181a	-2.917067207
hsa-miR-126	-2.917561364
hsa-miR-199a-5p	-2.955953091
hsa-miR-150	-2.965560902
hsa-miR-25	-3.000046266
hsa-miR-155	-3.012927327
hsa-miR-199a-3p	-3.022860966
hsa-miR-1228-star	-3.038080821
hsa-miR-361-5p	-3.045102941
hsa-miR-30c	-3.056454043
hsa-miR-92a	-3.065508171
hsa-miR-320c	-3.077622432
hsa-miR-584	-3.08166198
hsa-miR-130a	-3.138806298
hsa-let-7e	-3.153144421
hsa-miR-23b	-3.183528931
hsa-miR-638	-3.225527797
hsa-let-7f	-3.251514821
hsa-miR-27a-star	-3.350893013
hsa-miR-199b-3p	-3.353999561
hsa-miR-320d	-3.384660486
hsa-let-7b	-3.39766532
hsa-miR-197	-3.452295901
hsa-miR-221	-3.4672296
hsa-miR-1308	-3.502713525
hsa-miR-151-5p	-3.559191665
hsa-miR-223	-3.583694421
hsa-miR-15b	-3.594455829

hsa-miR-1268	-3.623250682
hsa-let-7d	-3.660578086
hsa-miR-23a-star	-3.674395381
hsa-miR-145	-3.702196255
hsa-miR-1281	-3.7157141
hsa-let-7c	-3.909288006
hsa-miR-1826	-4.006793406
hsa-let-7a	-4.007043371
hsa-miR-149-star	-4.138447771
hsa-miR-1207-5p	-4.869370833
hsa-miR-923	-7.618331152

Supplementary Table S5: List of significantly altered miRNAs in PTSD and their predicted target genes (present in our RNA-Seq dataset) obtained after Expression pairing in Ingenuity Pathway Analysis (IPA).	
**miRNA ID**	**Target gene ID (present in the RNA-Seq dataset)**
hsa-let-7a	NM_006988, NM_145236, NM_030806, NM_058229, NM_001145770
hsa-miR-103	NM_000491, NM_012317, NM_014840
hsa-miR-1207-5p	NM_145236, NM_000491, NM_018397, NM_002089, NM_002090, NM_001145770, NM_144646, NM_007360, NM_138360, NM_001303012, NM_013364, NM_033256, NM_020431
hsa-miR-125a-5p	NM_006988, NM_027353, NM_000598, NM_005041, NM_015894
hsa-miR-1268	NM_000598
hsa-miR-128	NM_145236, NM_030806, NM_002089, NM_001023567, NM_144646
hsa-miR-139-5p	NM_001023567, NM_001145770
hsa-miR-140-5p	NR_027353, NM_004654
hsa-miR-146b-5p	NM_002985
hsa-miR-16	NM_006988, NM_030806, NM_000491, NM_012317, NM_014840
hsa-miR-181a	NM_006988, NM_001023567, NM_4654
hsa-miR-188-3p	NM_015894, NM_003394
hsa-miR-663	NR_027353, NM_012317, NM_173821, NM_052863, NM_015894, NM_013351, NM_020431
hsa-miR-324-3p	NM_001040025, NM_006039, NM_013364
hsa-miR-193a-5p	NM_001023567, NM_181786
hsa-miR-194	NM_030806 NM_002090, NR_024011
hsa-miR-199b-3p	NM_030806, NM_001765
hsa-miR-199a-5p	NM_030806, NM_004131
hsa-miR-19b	NM_058229, NM_001023567, NM_000598, NM_014840, NM_001008, NM_032108, NM_030753
hsa-miR-219-1-3p	NM_015167, NM_000891, NM_004668
hsa-miR-24	NM_018397, NM_001145770, NM_181786, NM_006039, NM_030760
hsa-miR-29b	NM_030806
	NR_024011
	NM_013351
hsa-miR-30c	NM_058229
	NM_014840
	NM_032108
hsa-miR-320d	NM_153259
	NM_003151
	NM_003394
hsa-miR-331-3p	NM_145236
	NM_058229
	NM_001178126
	NM_007360
	NM_014840
	NM_030760
hsa-miR-339-3p	NM_015894
hsa-miR-339-5p	NM_002090
	NM_001145770
	NM_000598
	NM_014840
	NM_013364

hsa-miR-342-3p	NM_145236
	NM_058229
	NM_003394
hsa-miR-342-5p	NM_145236
	NM_030806
	NM_030760
	NM_020431
hsa-miR-409-3p	NM_001303012
hsa-miR-423-5p	NM_145236
	NM_002985
	NM_001765
	NR_027353
	NM_002122
	NM_006039
	NM_005601
	NM_052863
hsa-miR-455-3p	NM_030806
	NM_005041
	NM_173799
hsa-miR-486-5p	NM_030806
hsa-miR-491-5p	NM_001145770
	NM_020431
hsa-miR-502-3p	NM_002122
hsa-miR-532-3p	NM_030806
	NM_002090
	NM_001008226
	NM_001023567
	NM_030760
hsa-miR-532-3p	NM_002089
	NM_007360
	NM_014840
hsa-miR-542-5p	NM_013364
hsa-miR-574-3p	NM_005601
hsa-miR-574-5p	NM_001135865
hsa-miR-615-5p	NM_001134194
	NM_001082
	NM_000896
	NM_001440
	NM_005242
	NM_019099
	NM_033393
	NM_052972
	NM_022746
	NM_024298
	NM_004668
	NM_004148
	NM_032536
	NM_007365
	NM_001002814
	NM_004283
	NM_014737

	NM_021202
hsa-miR-628-3p	NM_006988
hsa-miR-671-5p	NM_006988
	NM_015991
	NM_022555
	NM_032108
hsa-miR-28-5p	NM_014840
	NM_004654
hsa-miR-92a	NM_058229
	NM_001023567
	NM_153259

Supplementary Table S6: Genes from the RNA-Seq dataset and their DNA methylation level (Uddin et al., 2010) at the CpG sites near promoter						
			DNA methylation β value		Expressed in %	
gene_id	log2 (FC)	Illumina ID for CpG islands	Control	PTSD	Control %	PTSD %
NM_015714	-3.77249	cg08185241	0.10664	0.115408	10.66399	11.54077
NM_033655	-3.45161	cg13059782	0.107877	0.110226	10.78768	11.02263
NM_005581	-3.22104	cg21263196	0.040464	0.057431	4.046352	5.743132
NM_000478	-3.15304	cg20645065	0.045869	0.080185	4.58686	8.018517
NM_001124	-2.71289	cg12320306	0.029072	0.045684	2.907244	4.568398
NM_006018	-2.41909	cg05384917	0.878164	0.893789	87.81641	89.3789
NM_005306	-2.41673	cg15479752	0.897454	0.930961	89.74544	93.09607
NM_002155	-2.38861	cg24192663	0.624213	0.65918	62.42135	65.91805
NM_002993	-2.37093	cg25432696	0.082599	0.111331	8.259885	11.13312
NM_002514	-2.26268	cg20379125	0.024985	0.030035	2.498505	3.003514
NM_052839	-2.21281	cg01606998	0.059053	0.064606	5.905284	6.460622
NM_006167	-2.11232	cg21481775	0.07202	0.11019	7.202008	11.019
NM_006705	-2.06904	cg21912567	0.032268	0.054409	3.226839	5.440854
NM_024565	-2.0358	cg17178888	0.036163	0.040318	3.616259	4.031796
NM_004664	-1.90634	cg05647859	0.136636	0.146246	13.66365	14.62459
NM_012337	-1.89692	cg02849695	0.408888	0.437582	40.88878	43.7582
NM_005091	-1.87153	cg02806777	0.201115	0.237597	20.11154	23.75968
NM_006317	-1.84228	cg23496260	0.049395	0.056596	4.939471	5.659572
NM_033027	-1.83734	cg02254461	0.790954	0.820824	79.09544	82.0824
NM_014326	-1.77918	cg23165541	0.726448	0.739955	72.64481	73.99547
NM_002108	-1.71994	cg27246571	0.72369	0.749337	72.36898	74.93369
NM_003043	-1.56557	cg13763232	0.822652	0.847083	82.2652	84.70826
NM_000167	-1.53498	cg15636587	0.19244	0.235275	19.24397	23.52748
NM_021083	-1.5015	cg07489003	0.750652	0.779277	75.0652	77.92765
NM_004536	-1.45422	cg01673583	0.843072	0.868505	84.30718	86.85053
NM_138788	-1.44668	cg05067286	0.243833	0.268424	24.3833	26.84241
NM_002863	-1.4257	cg22960284	0.04503	0.057896	4.503004	5.789611
NM_006456	-1.41191	cg21522303	0.031552	0.049133	3.1552	4.9133
NM_004049	-1.39153	cg24924631	0.63864	0.643363	63.864	64.3363
NM_080387	-1.34041	cg09546307	0.628535	0.667703	62.8535	66.7703
NM_024617	-1.33553	cg05065037	0.046022	0.056873	4.6022	5.6873
NM_014957	-1.26401	cg16425577	0.077807	0.090274	7.78069	9.027427
NM_031476	-1.25072	cg07207789	0.681477	0.710524	68.14772	71.05239
NM_001150	-1.2071	cg05985767	0.861835	0.879598	86.18348	87.95978
NM_148962	-1.17745	cg23759710	0.545449	0.562017	54.54492	56.2017
NM_003749	-1.11358	cg25802424	0.02411	0.034692	2.410999	3.469228
NM_173822	-1.10926	cg14047656	0.026775	0.032799	2.677476	3.279856
NM_003174	-1.07427	cg03259469	0.048204	0.06542	4.820393	6.542007
NM_022746	-1.00704	cg02265318	0.050013	0.063341	5.00131	6.334149

NM_004283	-1.00505	cg20780953	0.062019	0.09422	6.2019	9.422
			**DNA methylation β value**		**Expressed in %**	
**gene_id**	**log2 FC**	**Illumina ID for CpG islands**	**Control**	**PTSD**	**Control %**	**PTSD %**
NM_058229	1.00345	cg25406735	0.023595	0.011652	2.3595	1.1652
NM_003151	1.06681	cg20195812	0.067109	0.055412	6.710918	5.54115
NM_003394	1.15246	cg05164634	0.036298	0.028078	3.629845	2.807764
NM_001975	1.1608	cg13334990	0.25642	0.230102	25.64202	23.01024
NM_004669	1.18195	cg15387123	0.079	0.070573	7.899981	7.0573
NM_030760	1.20954	cg24807354	0.063902	0.05765	6.390206	5.764969
NM_006988	1.46005	cg00472814	0.036104	0.027095	3.610413	2.709523
NM_000598	1.68741	cg22083798	0.087192	0.078724	8.719159	7.872437
NM_014840	1.96002	cg23555120	0.155161	0.120884	15.51612	12.08836
NM_018397	2.00633	cg12991365	0.224253	0.185049	22.42525	18.50489
NM_052863	2.54427	cg14472601	0.561465	0.521927	56.1465	52.19265
NM_030753	2.83619	cg00126034	0.032501	0.021376	3.250058	2.137591
